# Symptomatic severe acute respiratory syndrome coronavirus 2 reinfection in a lupus patient treated with hydroxychloroquine: a case report

**DOI:** 10.1186/s13256-021-03159-9

**Published:** 2021-11-26

**Authors:** Astrid Muyldermans, Piet Maes, Tony Wawina-Bokalanga, Tine Anthierens, Olivier Goldberg, Magali Bartiaux, Oriane Soetens, Ingrid Wybo, Sigi Van den Wijngaert, Denis Piérard

**Affiliations:** 1 Department of Microbiology and Infection Control, Vrije Universiteit Brussel (VUB), Universitair Ziekenhuis Brussel (UZ Brussel), Laarbeeklaan 101, 1090 Brussels, Belgium; 2 Department of Microbiology, Immunology and Transplantation, Laboratory of Clinical and Epidemiological Virology, KU Leuven, Rega Institute for Medical Research, Leuven, Belgium; 3 Department of Emergency Medicine, Vrije Universiteit Brussel (VUB), Universitair Ziekenhuis Brussel (UZ Brussel), Brussels, Belgium; 4 Department of Emergency Medicine, Centre Hospitalier Universitaire Saint-Pierre (CHUSP), Brussels, Belgium; 5 Department of Microbiology, Laboratoire Hospitalier Universitaire Bruxelles-Universitair Laboratorium Brussel (LHUB-ULB), Brussels, Belgium

**Keywords:** SARS-CoV-2, COVID-19, Reinfection, Hydroxychloroquine, Lupus, Case report

## Abstract

**Background:**

Hydroxychloroquine and chloroquine have been used for hospitalized coronavirus disease 2019 patients because of their antiviral and anti-inflammatory function. However, little research has been published on the impact of the immunomodulatory effect of (hydroxy)chloroquine on humoral immunity.

**Case presentation:**

We report a case of symptomatic severe acute respiratory syndrome coronavirus 2 reinfection, diagnosed 141 days after the first episode, in a 56-year-old man of Black African origin treated with hydroxychloroquine for lupus erythematosus. No anti-severe acute respiratory syndrome coronavirus 2 IgG antibodies could be detected 127 days after the initial episode of coronavirus disease 2019.

**Conclusions:**

The treatment with hydroxychloroquine probably explains the decreased immune response with negative serology and subsequent reinfection in our patient. As humoral immunity is crucial to fight a severe acute respiratory syndrome coronavirus 2 infection, the use of (hydroxy)chloroquine is likely to have a detrimental effect on the spread of the virus. This case emphasizes that more needs to be learned about the role of antibodies in protecting against severe acute respiratory syndrome coronavirus 2 (re)infection and the role of (hydroxy)chloroquine on humoral immunity.

## Background

(Hydroxy)chloroquine has been used for decades as prophylaxis and treatment of malaria and autoimmune diseases such as lupus erythematosus. In March 2020, the US Food and Drug Administration (FDA) allowed the use of hydroxychloroquine and chloroquine for certain hospitalized coronavirus disease 2019 (COVID-19) patients as an emergency use authorization (EUA). Possible beneficial effects may be attributed to its antiviral and anti-inflammatory function [1, 2]. In June 2020, this EUA was revoked as the known and potential benefits no longer outweighed the known and potential risks, including serious cardiac adverse events. However, little research has been published on the impact of the immunomodulatory effect of (hydroxy)chloroquine on humoral immunity [1, 3].

## Case presentation

**Fig. 1:**
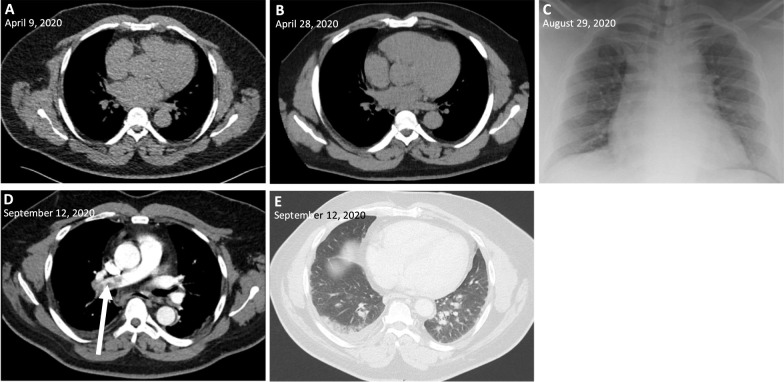
Chest computed tomography and radiograph images during the disease course. **A**–**B**, chest computed tomography (CT) without abnormalities; **C** bedside chest radiograph without alveolar consolidations; **D** CT pulmonary angiogram showing pulmonary embolism (arrow); **E** chest CT showing peripheral ground-glass opacities and pleural effusion in the right lower lobe

On 9 April 2020, a 56-year-old obese man (BMI 35) of Black African origin with discoid lupus erythematosus (treated with hydroxychloroquine 200 mg twice a day) presented at the emergency department (ED) of the Centre Hospitalier Universitaire Saint-Pierre (CHUSP) with dyspnea for 2 weeks, dry cough, chest pain, myalgia, headache, ageusia, and diarrhea. One week earlier he had returned from the Democratic Republic of Congo (DRC) where he resided for 2 months. Malaria prophylaxis (atovaquone/proguanil) was taken correctly. Upon admission, a nasopharyngeal swab was taken and severe acute respiratory syndrome coronavirus 2 (SARS-CoV-2) was detected by real-time reverse transcription polymerase chain reaction (RT-PCR) (RealStar^®^ SARS-CoV-2 RT-PCR Kit 1.0, Altona Diagnostics GmbH, Hamburg, Germany; targeting E-gene and S-gene) with a crossing point (Cp) of 36, but no abnormalities were observed on a chest computed tomography (CT) (Fig. 1) and oxygen saturation was 100%. His body temperature was 37.1 ℃ and the following laboratory parameters were within normal limits: C-reactive protein (CRP; 1.0 mg/L), leukocytes (4.3 × 10^3^/μL), lymphocytes (1.8 × 10^3^/μL), neutrophils (1.8 × 10^3^/μL), platelets (214 × 10^3^/μL), and hemoglobin (14.7 g/dL). The patient was placed in home quarantine for 2 weeks.

On 28 April he presented himself to Universitair Ziekenhuis Brussel's (UZB’s) ED with chest pain, abdominal pain, and diarrhea for a week. The imaging was repeated, but again no abnormalities were observed on a chest CT (Fig. 1). However, cardiac troponin T was slightly elevated (0.011 μg/L), suggesting the diagnosis of pericarditis. Aspirin (1000 mg four times a day) was prescribed and the patient was discharged.

On 4 June, SARS-CoV-2 RT-PCR (RealStar®) was performed prior to a planned 1-day hospitalization in UZB for gastroscopy because of postprandial bloating. This test was negative. No abnormalities were found by the gastroscopy, and the patient decided to discontinue hydroxychloroquine on his own initiative as gastrointestinal discomfort can be a side effect of the drug.

A serological analysis (LIAISON® SARS-CoV-2 S1/S2 IgG, Diasorin, Saluggia, Italy) performed 127 days after the initial episode (14 August) could not detect anti-SARS-CoV-2 IgG antibodies against spike protein.

On 28 August (141 days after the initial episode), the patient presented at UZB’s ED with dyspnea, productive cough, malaise, fever, dysosmia, and dysgeusia for 3 days. A nasopharyngeal swab was taken showing a strong positive result for SARS-CoV-2 (Cp 14) (RealStar®). Moreover, laboratory analysis showed a mild leukopenia (3.2 × 10^3^/μL) and lymphocytopenia (0.9 × 10^3^/μL), however CRP (1.8 mg/L), neutrophils (1.6 × 10^3^/μL), platelets (168 × 10^3^/μL), and hemoglobin (13.2 × g/dL) were within normal limits. No abnormalities were observed on a bedside chest radiograph (Fig. 1). The patient was placed in home quarantine, but presented himself again 4 days later (1 September) because of persistent complaints with decreased oxygen saturation (93.9%). He was hospitalized and oxygen therapy was started (2 L/min). The oxygen could be stopped after 12 hours (oxygen saturation 99%) and he stayed in the hospital for 1 week with symptomatic treatment. An evaluation for humoral immune deficiency was performed, however no general antibody deficiency was observed by measurement of serum immunoglobulin levels (IgG, IgA, IgM). Only 4 days after his hospital discharge, the patient presented at CHUSP’s ED with dyspnea, productive cough, and chest pain. Laboratory results showed an elevation of D-dimer (4183 ng/mL) and CRP 32 mg/L. The following parameters were within normal limits: leukocytes (8.3 × 10^3^/µL), lymphocytes (1.9 × 10^3^/µL), neutrophils (5.4 × 10^3^/µL), platelets (314 × 10^3^/µL), and hemoglobin (13.0 g/dL). A CT pulmonary angiogram was performed showing pulmonary embolism and ground-glass opacities compatible with viral pneumonia (Fig. 1). Serological analysis performed 158 days after the first episode (14 September), showed the presence of anti-SARS-CoV-2 IgG antibodies to spike protein (130 AE/mL, Diasorin). Anticoagulation by tinzaparin sodium was initiated (followed by rivaroxaban after 14 days, 15 mg twice a day), as well as empirical antibiotic therapy by ceftriaxone (2 g daily for 7 days). No antiviral therapy or supplemental oxygen was started. On 29 September he was discharged, 173 days after the initial episode.

Genome sequencing was performed on nasopharyngeal swabs from the first and second episode with a MinION (Oxford Nanopore Technologies, Oxford, United Kingdom) using the ARTIC network nCoV-2019 sequencing protocols and analytic pipeline by Josh Quick [4]. From the sample of the initial episode, taken 2 weeks after the first symptoms, only a fragmented genome (6028 out of 29903 bps) could be determined, most likely due to a low viral load of the nasopharyngeal swab (Cp 36). A full-length sequence of the second episode could be determined, revealing a lineage B.1. SARS-CoV-2 [5].

Seven mutations were identified across the genome of the two strains (Table 1). Especially the key block mutation at positions 28881 to 28883 (AAC to GGG) in the nucleocapsid phosphoprotein region resulting in an amino acid change (lysine-arginine to arginine-glycine), indicates that the patient suffered from a reinfection [6]. The coverage for this triplet region was 121-fold for the first episode and 1586-fold for the second episode (with presence of the mutation in 100% of the reads).

**Table 1:** Observed mutations between the genomes of the first and second episode

Position (bp)	Base change	Gene
241	C to T	ORF1a
28831	C to T	Nucleocapsid phosphoprotein
28854	C to T	Nucleocapsid phosphoprotein
28881–28883	AAC to GGG	Nucleocapsid phosphoprotein
29034	A to C	Nucleocapsid phosphoprotein

## Discussion and conclusions

The patient was likely initially infected in the DRC, which counted 134 confirmed cases in the beginning of April 2020, as he already had symptoms during his stay [7]. The viral load tested on his return to Belgium was low. The patient experienced only mild symptoms during this first episode. At that moment, the patient was treated with hydroxychloroquine because of lupus. Since the EUA from FDA for (hydroxy)chloroquine for hospitalized COVID-19 patients, an increasing number of studies have been published with conflicting results about its effectivity [2, 8, 9]. However, little research has been published on the impact of the immunomodulatory effect of (hydroxy)chloroquine on humoral immunity [1, 3]. Chloroquine has been shown to suppress the antibody responses to vaccines against rabies, tetanus, and diphtheria [3]. This can be attributed to the fact that (hydroxy)chloroquine affects functions of proteins involved in antigen-presenting pathways and B-cell activation [3]. In a study of patients with Chikungunya virus infection, it was shown that the adaptive immune response was delayed due to chloroquine treatment in the acute phase [10]. So, hydroxychloroquine treatment may impair host immunity in response to SARS-CoV-2, however the effects on immune cell function have not been extensively examined [11]. As humoral immunity is crucial to fight a SARS-CoV-2 infection, the use of (hydroxy)chloroquine is likely to have a detrimental effect on the spread of the virus [1].

Our patient was treated with hydroxychloroquine for lupus, probably explaining the decreased immune response with negative serology (IgG) 127 days after the initial episode of COVID-19, and subsequent reinfection. At the time of reinfection, the use of hydroxychloroquine was discontinued and an IgG antibody response was detected 158 days after the initial episode. It has been shown that the IgG antibody response after a COVID-19 infection can wane with possible reinfection [12–14]. However, to conclude, our case emphasizes the need for trials about the role of COVID-19 treatment in general, and (hydroxy)chloroquine in particular, on the (humoral) immunity response [3].

## Data Availability

All data generated or analysed during this study are included in this published article.

## Abbreviations

FDA: Food and Drug Administration
EUA: Emergency use authorization
ED: Emergency department
DRC: Democratic Republic of Congo
SARS-CoV-2: Severe acute respiratory syndrome coronavirus 2
RT-PCR: Reverse transcription polymerase chain reaction
Cp: Crossing point
CT: Computed tomography
COVID-19: Coronavirus disease 2019
